# Croup

**Published:** 1883-12

**Authors:** 


### CROUP.

[From Dr. Hall's Health at Home.]

CROUP is a conjestion of blood in the arteries of the windpipe congested to such an extent that the more watery portions of the blood exude and spread and thicken, until the windpipe is so nearly closed that breathing is difficult, and as the filling-up increases and the breathing becomes more labored a kind of spasmodic contraction of the top of the windpipe takes place, and the child is dead.

Croup is the result of cold, especially is connected with damp clothing or wet stockings. No mother should ever put her child, under seven years, to bed without feeling the feet, and if they are not warm by all means warm them, as it may end in croup before morning.

Being out of doors after sundown from November to May is a very frequent cause of croup in small children ; in playing about their feet are apt to get wet, or they get over-excited in their little play, are overheated, and are very much inclined to stand in the wind, or at a corner on damp ground, or sit on a cold stone.

Croup usually comes on with a slight increase in the frequency of breathing, about sundown or bedtime. The next morning it seems to be better, and the mother is hopeful; but at night it is worse, and the third night or sooner it is the regular croup; the child is restless, uneasy, it breathes hard and fast, the chest heaves, there is a kind of wheezing, barking, suppressed cough, which does not seem to relieve.

If mothers would apply remedies the first night, croup is as easily cured as a common cold, when taken in time. The instant croupy threatenings are observed the child should be kept indoors; should eat very light food indeed, and not much of that, until the symptoms have abated. Hydropathists invest the throat immediately with cloths wet with water, very cold, ice-cold if possible, but not so as to dribble about; the wet cloth should be covered with a dry flannel one. These cloths should be renewed every two to ten minutes, according to the violence of the symptoms, to be continued, by all means, until the breathing is comfortably easy.

If there is not much fever, or if the skin is dry, put the child into a tepid bath of seventy-five degrees, and then well wrap up in a blanket until perspiration takes place. But if there is much fever and a hot skin use the wet pack sheet and renew until the fever abates. The bowels should be emptied at once with a warm water enema. By all possible means keep the feet and hands warm. If there is no expectoration, and the child seems to be almost suffocating, give warm water copiously, until the use of a feather to tickle the throat induces vomiting. It is sometimes wonderful to see the good effects of this warm water vomiting in cases of croup in children.

Some physicians consider nauseating remedies indispensable. Mix half a teaspoon each of powdered alum and ipecac in half a glass of tepid water, and give it as quickly as possible. If it does not cause the child to vomit in ten minutes repeat the dose, with a teacup of warm water every five minutes, until a feather or the finger in the throat produces copious vomiting.

